# P-2301. Predictors and clinical outcomes of hypogammaglobulinemia in liver transplant recipients: role of pre-transplant factors and graft volume

**DOI:** 10.1093/ofid/ofae631.2454

**Published:** 2025-01-29

**Authors:** Yongseop Lee, Eun-Ki Min, Juhan Lee, Deok-Gie Kim, Myoung Soo Kim, Jaeeun Seong, Sangmin Ahn, Min Han, Jung Ah Lee, Jung Ho Kim, Jin Young Ahn, Nam Su Ku, Jun Yong Choi, Joon-sup Yeom, Jae Geun Lee, Su Jin Jeong

**Affiliations:** Division of Infectious Diseases, Department of Internal Medicine and AIDS Research Institute, Yonsei University College of Medicine, Seodaemun-gu, Seoul-t'ukpyolsi, Republic of Korea; Department of Transplantation Surgery, Yonsei University College of Medicine, Seodaemun-gu, Seoul-t'ukpyolsi, Republic of Korea; Department of Transplantation Surgery, Yonsei University College of Medicine, Seodaemun-gu, Seoul-t'ukpyolsi, Republic of Korea; Department of Transplantation Surgery, Yonsei University College of Medicine, Seodaemun-gu, Seoul-t'ukpyolsi, Republic of Korea; Severance Hospital, Yonsei University College of Medicine, Seoul, Seoul-t'ukpyolsi, Republic of Korea; Division of Infectious Diseases, Department of Internal Medicine and AIDS Research Institute, Yonsei University College of Medicine, Seodaemun-gu, Seoul-t'ukpyolsi, Republic of Korea; Yonsei University College of Medicine, seoul, Seoul-t'ukpyolsi, Republic of Korea; Yonsei University School of Medicine, Seoul, Seoul-t'ukpyolsi, Republic of Korea; Yonsei University College of Medicine, seoul, Seoul-t'ukpyolsi, Republic of Korea; Yonsei University College of Medicine, seoul, Seoul-t'ukpyolsi, Republic of Korea; Yonsei University College of Medicine, seoul, Seoul-t'ukpyolsi, Republic of Korea; Division of Infectious Diseases, Department of Internal Medicine, Yonsei University College of Medicine, Seoul, Seoul-t'ukpyolsi, Republic of Korea; Yonsei University College of Medicine, seoul, Seoul-t'ukpyolsi, Republic of Korea; Division of Infectious Diseases, Department of Internal Medicine, Yonsei University College of Medicine, Seoul, Seoul-t'ukpyolsi, Republic of Korea; Department of Transplantation Surgery, Yonsei University College of Medicine, Seodaemun-gu, Seoul-t'ukpyolsi, Republic of Korea; Yonsei University College of Medicine, seoul, Seoul-t'ukpyolsi, Republic of Korea

## Abstract

**Background:**

Hypogammaglobulinemia (HGG) is a common complication after liver transplantation and is associated with severe bacterial infections and mortality. Although hepatocytes synthesize immunoglobulins, how underlying liver disease affects immunoglobulin dynamics after liver transplantation has not been well evaluated. We evaluated differences in serum levels of immunoglobulin G (IgG) depending on pre-transplant model for end-stage liver disease (MELD) score.

**Table 1.**
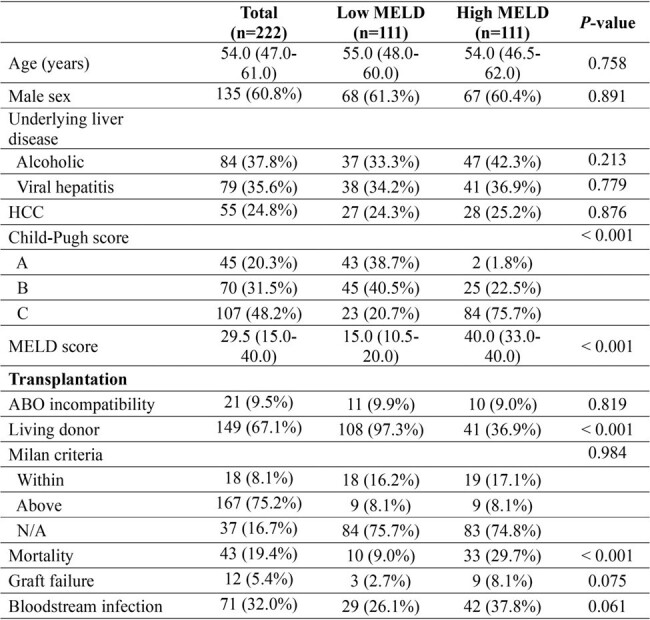
Characteristics of liver transplantation recipients after propensity score matching

**Methods:**

We prospectively enrolled patients who underwent liver transplantation between July 2016 and December 2022. Patients with pre-transplant MELD score < 30 and ≥30 were categorized as low and high MELD groups, respectively. Serum IgG levels were measured by enzyme-linked immunosorbent assay.**Figure 1.** Comparison of serum immunoglobulin G levels in liver transplant recipients between low and high MELD groups.

**Figure:**
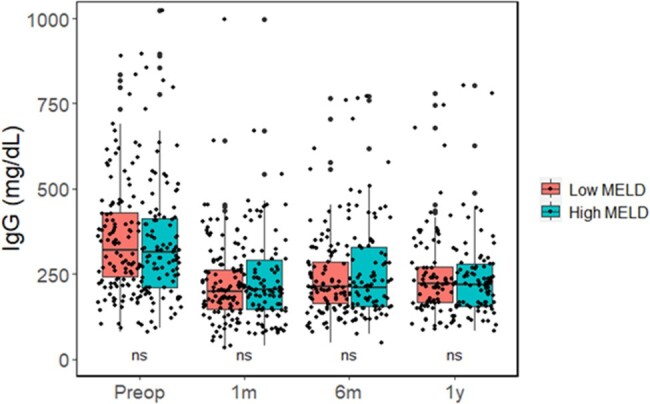
ns, not significant; *, P < 0.05; **; P < 0.01; ***, P < 0.001.

**Results:**

Of 559 liver transplant recipients, 444 were in the low MELD group and 111 were in the high MELD group. Propensity score matching was conducted between the two groups. The median serum IgG levels decreased significantly from 319.7 mg/dL pre-transplant to 201.3 mg/dL post-transplant at 1 month. The IgG levels at pre-transplant and post- transplant (1 month, 6 months, and 1 year) did not differ significantly between the low and high MELD groups. High MELD (adjusted hazard ratio [aHR], 3.71; 95% confidence interval [CI], 1.64–8.40; P = 0.002) and pre-transplant HGG (aHR, 2.16; 95% CI, 1.05–4.46; P = 0.037) were independently associated with overall mortality. In addition, hepatocellular carcinoma (aHR, 2.08; 95% CI, 1.07–4.03; P = 0.031), high MELD (aHR, 1.85; 95% CI, 1.01–3.37; P = 0.046) and post-transplant HGG (aHR, 2.35; 95% CI, 1.12–4.95; P = 0.023) were associated with bloodstream infections. Predictors for HGG were viral hepatitis (odds ratio [OR], 0.46; 95% CI 0.22­–0.90; P = 0.027) for pre-transplant, and hepatocellular carcinoma (OR, 0.34; 95% CI 0.12–0.79); P = 0.020) and graft to recipient weight ratio ≥ 0.8 (OR 0.26; 95% CI 0.07–0.90; P = 0.033) for post-transplant.

**Table 2.**
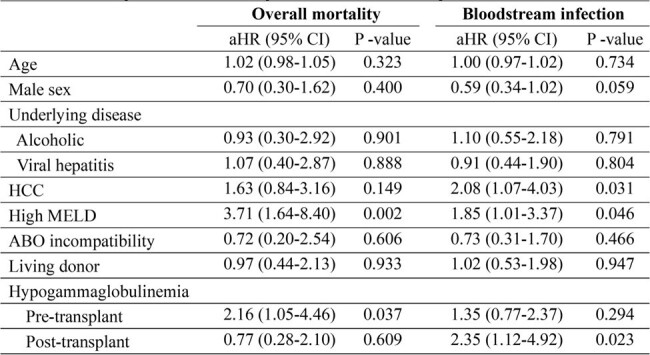
Multivariable cox proportional regression of effect of hypogammaglobulinemia in liver transplantation recipients on mortality and bloodstream infection.

Hypogammaglobulinemia was defined as the lowest quartile of serum immunoglobulin G levels. Post-transplant hypogammaglobulinemia was assessed at post-operative 1 month. Post-transplant hypogammaglobulinemia was used as a time-varying variable.

**Conclusion:**

Peri-transplant HGG was associated with poor outcomes including mortality and severe infections. There is no significant difference in HGG according to MELD score, however, it is associated with insufficient graft volume.

**Table 3.**
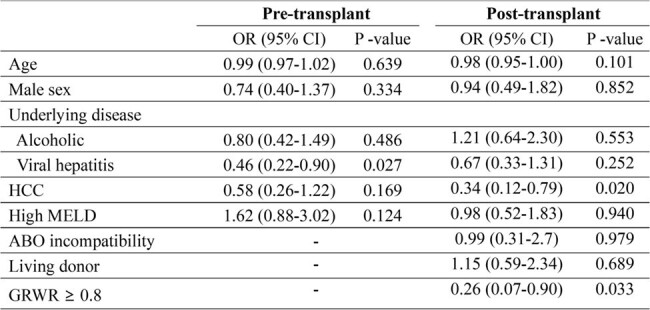
Univariable analysis for hypogammaglobulinemia in liver transplantation recipients

Hypogammaglobulinemia was defined as the lowest quartile of serum immunoglobulin G levels. Post-transplant hypogammaglobulinemia was assessed at postoperative 1 month.

**Disclosures:**

All Authors: No reported disclosures

